# Breakfast Skipping among a Multi-Ethnic Population of Young Men and Relationship with Sociodemographic Determinants and Weight Status

**DOI:** 10.3390/ijerph19052903

**Published:** 2022-03-02

**Authors:** Jozaa Z. AlTamimi, Naseem M. Alshwaiyat, Hana Alkhalidy, Nora A. AlFaris, Nora M. AlKehayez, Reham I. Alagal

**Affiliations:** 1 Department of Physical Sports Sciences, College of Education, Princess Nourah bint Abdulrahman University, P.O. Box 84428, Riyadh 11671, Saudi Arabia; jzaltamimi@pnu.edu.sa (J.Z.A.); naalfaris@pnu.edu.sa (N.A.A.); nmalkehayez@pnu.edu.sa (N.M.A.); 2 School of Nutrition and Dietetics, Faculty of Health Sciences, Gong Badak Campus, Universiti Sultan Zainal Abidin, Kuala Nerus 21300, Terengganu, Malaysia; sh_naseem@yahoo.com; 3 Department of Nutrition and Food Technology, Faculty of Agriculture, Jordan University of Science and Technology, Irbid 22110, Jordan; haalkhalidy@just.edu.jo

**Keywords:** breakfast skipping, multi-ethnic, young men, weight status, body mass index

## Abstract

Breakfast skipping is linked with obesity incidence. This study was conducted to assess the prevalence of breakfast skipping among a multi-ethnic population of young men residing in Saudi Arabia and its relationship with sociodemographic determinants and weight status. A total of 3600 young men aged 20 to 35 years and living in Riyadh, Saudi Arabia, were involved in this cross-sectional study. Sociodemographic determinants and breakfast-consumption frequency were collected from subjects by personal interviews. This study defines breakfast skipping as skipping breakfast at least one day per week. Weight and height were measured following standardized methods. The prevalence of breakfast skipping was observed among 52.8% of the study subjects. Nationality was a predictor of breakfast skipping, with the lowest and highest rates of breakfast skipping reported among young men from Bangladesh (14.0%) and Saudi Arabia (86.5%), respectively. Weight status was another predictor of breakfast skipping, as the mean body mass index for breakfast skippers (25.4 kg/m^2^) was significantly (*p*-value < 0.001) higher than that for breakfast consumers (24.8 kg/m^2^). Overweight/obese subjects have a significantly higher rate of breakfast skipping (56.9%) than underweight/normal weight subjects (48.9%). In conclusion, breakfast skipping prevalence is relatively high among young men residing in Saudi Arabia. The findings confirm a relationship between breakfast skipping and sociodemographic determinants and weight status.

## 1. Introduction

Overweight and obesity rates are continuously rising at the global level [1]. Obesity is the most common form of malnutrition and is linked to the elevated prevalence of chronic diseases, nutritional deficiencies, and worldwide disease burden [2,3,4]. Several eating patterns, such as frequency of meals eaten during the day, eating at night, the portion size of meals, frequency of meals eaten outside the home, and breakfast skipping, are closely connected with obesity incidence [5,6,7,8]. Breakfast is defined differently in different studies. However, breakfast is commonly known as the foremost meal during the day after waking up in the morning [9]. Breakfast is a crucial meal, as it provides the body with calories and essential nutrients after a long period of fasting during sleeping hours in the night [10].

Usually, breakfast skipping is associated with higher energy consumption during later meals and poor food quality [11]. Accumulating evidence indicates that breakfast skipping causes overcompensation for energy intake during the next meals throughout the day [12]. Breakfast consumption promotes higher food satiety in subsequent meals than does skipping breakfast. Likewise, a growing body of evidence reveals that breakfast skipping is connected with lower food quality [13]. Breakfast intake has been linked to better daily micronutrient intakes and food quality among adults [14]. Many studies found that eating more energy at breakfast was linked to an improved nutrients intake, healthier food choices throughout the day, and lower total energy intakes [15]. Actually, obtaining a higher fraction of total energy intake from breakfast is associated with higher intakes of protein, dietary fibers, vitamins, and minerals and lower intakes of saturated fat and added sugar. Contrarily, breakfast skipping is linked with lower food quality characterized by a high intake of total fat, saturated fat, and added sugar and a low intake of vitamins and minerals [11]. The negative impact of breakfast skipping on food quality could be because people typically consume particular foods at certain meals. For example, dairy and whole grains are usually eaten at breakfast. Thus, skipping breakfast may be affecting their consumption. Moreover, the hunger caused by skipping breakfast could shift the food choices of people at next meals toward energy and fat-dense foods [12].

Over the last few decades, the dietary behaviors of people have been changed markedly as a result of shifts in their daily lifestyles. Therefore, breakfast skipping has become more common among children, teenagers, and adults [16,17]. Young adulthood is a challenging life stage characterized by adopting many unhealthy dietary practices. One of these unhealthy dietary patterns commonly seen among young adults is breakfast skipping [18]. Breakfast skipping has become a contentious public health topic in recent years. Several efforts have been dedicated to studying the link between breakfast skipping and weight status. Yet, the findings of these reports are inconsistent. Many studies reported that breakfast skipping accompanies a rise in the incidence of obesity and obesity-related diseases, while eating breakfast can effectively lower obesity risk [19,20,21]. Nevertheless, some studies indicate an absence of connection between breakfast skipping and obesity, while others suggested that breakfast skipping could lead to body weight loss [22,23]. Furthermore, breakfast skipping was linked with an elevated risk of many diseases, such as depression, cardiovascular diseases, diabetes mellitus, and some cancers [24,25,26,27].

Saudi Arabia is a leading oil producer globally and has a fast-growing economy. As a result, employees from all over the world, primarily from the Middle East, South Asia, and Southeast Asia, come to work in Saudi Arabia. Expatriates in Saudi Arabia made up about half of the overall workforce and about 90% of the private sector employment in 2013 [28]. Non-Saudi residents formed about one-third of the population in this country; about three-fourths of them were males [29]. Migrants from various ethnic backgrounds provide an interesting opportunity to study differences in eating habits and their association with health and disease in a diverse population. Therefore, the current study was carried out to assess breakfast skipping among a multi-ethnic population of young men living in Saudi Arabia and its relationship with sociodemographic determinants and weight status.

## 2. Methods

### 2.1. Study Design and Participants

This study is part of a research project called the Relationship between Obesity, physical Activity, and Dietary pattern among men in Saudi Arabia (ROAD-KSA). This research project is a cross-sectional study designed to assess the rate of overweight and obesity, physical activity status, and dietary patterns among young and middle-aged men from twelve Middle Eastern and Asian countries living in Riyadh, Saudi Arabia, and the relationships between these variables. This study was carried out in Riyadh, Saudi Arabia.

Study participants were recruited randomly from public places in Riyadh city, such as public gardens and shopping malls, using a stratified clustered sampling technique based on geographic locations in the city. The eligible participants were young men (20 to 35 years) residing in Riyadh, free of any physical malfunction, and having a single nationality of one of the following countries: Saudi Arabia, Egypt, Yemen, Syria, Jordan, Sudan, Turkey, Pakistan, Afghanistan, India, Bangladesh, and the Philippines. In this study, being free of any physical malfunction is defined as an individual without any type of disability or any physical or mental condition that significantly limits motor, sensory, or cognitive abilities. The evaluation of this criterion was done by the researchers using a specific form that includes a list of conditions considered a disability. Therefore, volunteers who were free of all these conditions were considered eligible to participate in the current study. Participants signed a consent form prior to taking part in this study in harmony with the Helsinki Declaration. The research ethics committee of Princess Nourah bint Abdulrahman University approved this study.

### 2.2. Sociodemographic Determinants

Trained research assistants collected sociodemographic determinants using personal interviews. The collected sociodemographic determinants include the participants’ nationality, age, period of residence in Saudi Arabia, household type, marital status, educational level, and monthly income.

### 2.3. Measurements

Trained research assistants measured the body weight and height of participants. While wearing little clothing and without shoes, a calibrated digital weight scale was used to measure the body weight to the nearest 0.1 kg. A calibrated portable stadiometer was also used to measure the height in complete standing posture without shoes to the nearest 0.1 cm. The body mass index (BMI) was calculated by dividing weight in kilogram by height in meter square. Participants were categorized based on their BMI to underweight (<18.5), normal weight (18.5–24.9), overweight (25–29.9), or obese (≥30) [30].

### 2.4. Breakfast Consumption

Breakfast-consumption frequency was assessed by using a valid and reliable questionnaire. The face validity of the questionnaire was evaluated using an independent opinion from five experts in nutrition research. A test-retest pilot study with a two-week gap was carried out to assess the reliability of the questionnaire. Pilot study data were collected from 60 subjects from the target population and were not included in the study sample. All data collection was done by trained research assistants using personal interviews. The frequency of breakfast consumption was measured by asking the participants about the number of days per week they usually ate breakfast during the previous year. Responses ranged from zero to seven days per week. Breakfast is defined as any food or beverage eaten in the morning after waking up between 5:00 am and 11:00 am [31]. Breakfast skipping is defined as not eating breakfast one day or more per week, which is also used in previous studies [32,33,34,35,36].

### 2.5. Statistical Analysis

IBM SPSS Statistics for Windows (version 26. Armonk, NY, USA, 2019) was used for data analysis. Categorical variables were analyzed using the chi-square test and presented as numbers and percentages. Continuous variables were analyzed using independent samples *t*-test and presented as means and standard deviations. Univariate and multivariate logistic regression analyses were performed to detect the factors related to breakfast skipping. All *p*-values were obtained based on two-tailed tests. Differences were considered statistically significant at *p*-values < 0.05.

## 3. Results

**Figure 1:**
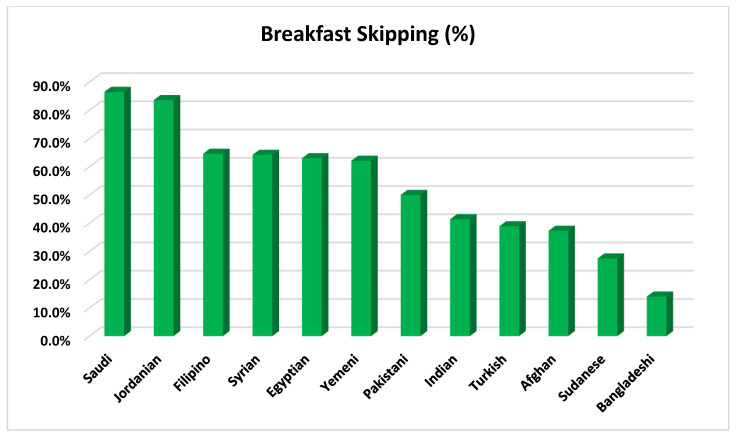
Bar chart illustrating breakfast-skipping prevalence among study participants stratified based on their nationality.

A total of 3600 participants were involved in the current study. The sociodemographic determinants and body weight status of the study participants stratified according to breakfast eating patterns are presented in Table 1. Breakfast skipping was reported among 52.8% of study participants. By nationality, the lowest and highest rates of breakfast skipping were reported among subjects from Bangladesh (14.0%) and Saudi Arabia (86.5%), respectively (Figure 1). Relatively high rates of breakfast skipping were seen among participants from Egypt (63.0%), Yemen (62.1%), Syria (64.2%), Jordan (83.6%), Pakistan (50.0%), and the Philippines (64.6%). However, relatively lower rates of breakfast skipping were reported among participants from Sudan (27.5%), Turkey (38.9%), Afghanistan (37.3%), and India (41.4%). Moreover, the mean age for breakfast skippers (29.3 ± 3.2) was significantly lower than that for breakfast consumers (29.9 ± 3.2). About half of participants with one to five years (51.5%) and six years or more (54.7%) of residency periods in Saudi Arabia were breakfast skippers. Participants living within family households have a significantly higher breakfast-skipping rate (69.1%) than those living within non-family households (49.0%). Similarly, single participants have a significantly higher breakfast-skipping rate (58.9%) than married participants (45.8%). Surprisingly, highly educated participants with at least a college degree have a significantly higher breakfast-skipping rate (69.5%) than less educated participants with high school or less (43.2%). Subjects with high monthly income (≥USD 1000) have a significantly higher breakfast-skipping rate (69.9%) than subjects with lower monthly income (46.5%). The mean BMI for breakfast skippers (25.4 ± 3.4) was significantly higher than that for breakfast consumers (24.8 ± 3.0). Overweight/obese subjects have a significantly higher breakfast-skipping rate (56.9%) than underweight/normal weight subjects (48.9%).

The odds ratios of breakfast skipping among all subjects for sociodemographic determinants and BMI are shown in Table 2. Compared with subjects from Bangladesh, who have the lowest breakfast-skipping rate, subjects from other countries had significantly higher odds ratios of being breakfast skippers, including Saudi Arabia (adjusted odds ratio (OR) = 27.19, *p* = 0.001), Egypt (adjusted OR = 8.80, *p* = 0.001), Yemen (adjusted OR = 7.79, *p* = 0.001), Syria (adjusted OR = 9.22, *p* = 0.001), Jordan (adjusted OR = 29.10, *p* = 0. 001), Sudan (adjusted OR = 2.23, *p* = 0.001), Turkey (adjusted OR = 3.58, *p* = 0.001), Pakistan (adjusted OR = 4.92, *p* = 0.001), Afghanistan (adjusted OR = 3.02, *p* = 0.001), India (adjusted OR = 4.10, *p* = 0.001), and the Philippines (adjusted OR = 10.12, *p* = 0.001). Moreover, increasing age was significantly associated with a lower odds ratio of breakfast skipping (adjusted OR = 0.97, *p* = 0.044). Married subjects had a significantly lower odds ratio of breakfast skipping compared with single subjects (adjusted OR = 0.63, *p* = 0.001). Participants having high monthly income (USD 1000 or more) had a significantly higher odds ratio of breakfast skipping compared with those who have low monthly income (adjusted OR = 1.37, *p* = 0.004). Finally, increasing BMI significantly accompanied higher breakfast-skipping odds ratio (adjusted OR = 1.03, *p* = 0.011).

Furthermore, subjects living within a family household had a significantly higher odds ratio of breakfast skipping than those living within a non-family household (unadjusted OR = 2.33, *p* = 0.001). Participants with a college degree or more had a significantly higher odds ratio of breakfast skipping than those with lower education levels (unadjusted OR = 2.99, *p* = 0.001). However, these associations were not confirmed in multivariate analysis.

## 4. Discussion

The present study investigated breakfast skipping among a multi-ethnic population of young men from twelve Middle Eastern and Asian countries living in Saudi Arabia. Our findings revealed that more than half of the participants (52.8%) were breakfast skippers. Several studies have investigated the prevalence of breakfast skipping among adults. A study from Saudi Arabia reported that 49.9% of male college students aged 18–24 years eat breakfast daily, while 50.1% skip breakfast at least once per week [37]. Data collected from four National Health and Nutrition Examination Surveys (NHANES) revealed that 26.1% and 21.2% of American young men and women aged 20–39 years were breakfast skippers, respectively [38]. A population-based study among Iranian university students found 47.8% of males and 43.8% of females skip eating breakfast at least three days per week [39]. Serbian National Health Survey data reported that 77.8 % of adults eat breakfast every day, while only 22.2% are breakfast skippers [6]. A study from Sweden found that 90% of adults aged 25–74 years usually consumed breakfast, while only 10% skipped breakfast [7]. Another study from Spain indicated that breakfast skipping was seen only among 4.5% and 2.3% of men and women aged 25–64 years, respectively [8]. A population-based study from Japan revealed that breakfast skipping was reported among 23.1% of adults aged 20–75 years (26.7% of men and 17.1% of women) [40]. Finally, data from Taiwan stated that breakfast skipping was reported among 49.7% and 50.3% of adult males and females [41].

Our results showed high variability in the prevalence of breakfast skipping among participants from different countries or ethnic backgrounds. This finding is consistent with several previous studies. A cross-sectional study that collected breakfast-consumption data of adults from eight European countries reported that breakfast-skipping rates differed significantly across countries [33]. While breakfast skipping rates were relatively high among participants from Greece (54%), Slovenia (49%), Hungary (43%), and Switzerland (34%), the rates were relatively low among participants from Belgium (18%), Norway (16%), Spain (14%), and the Netherlands (12%) [33]. In another study from the United States, breakfast skipping varied based on ethnicity. Breakfast skipping was reported among 23%, 31.8%, and 19.5% of White, Black, and Hispanic young American adults, respectively [38]. The current study found associations between breakfast skipping and age, household type, marital status, educational level, and monthly income. Current findings were consistent with results from earlier studies that reported that breakfast skippers have significantly lower age than breakfast consumers [38,39,40], and single adults have a significantly higher rate of breakfast skipping than married adults [38,39]. However, our results contrast findings from previous studies that found that breakfast skipping was significantly higher among participants with low education than those with high education [33] and participants with low income compared with those with high income [18,42]. The finding that participants with higher income had a higher odds ratio of breakfast skipping could be explained by the higher obesity prevalence observed among men with high income. In Saudi Arabia, financially stable men usually have office-type jobs reflecting low physical activity levels and a sedentary lifestyle [43]. This makes them more susceptible to obesity and affects their dietary behavior by skipping breakfast to lose weight.

Interestingly, our findings supported the associations between breakfast skipping and weight status. The relationship between breakfast skipping and weight status was proven among participants of different ages, genders, and counties [20]. These findings imply a worldwide association between skipping breakfast and weight status independent of these variables [20]. A meta-analysis study concluded that breakfast skipping is accompanied by a 75% higher obesity risk than breakfast consumption [17]. Another meta-analysis study confirmed that breakfast skipping is associated with weight status. This study concludes that breakfast skipping raised overweight and obesity risk by 48% based on data from cross-sectional studies and 44% based on data from cohort studies [20]. Numerous cohort studies demonstrated the link between breakfast skipping and weight status. A retrospective cohort study from Japan stated that breakfast skipping was accompanied by a more than 5% increase in BMI in male college students after one year of follow-up [44]. A five-year cohort of Australian young adults (26–36 years) concluded that weight gain was higher among breakfast skippers [45]. A longitudinal population-based cohort study from the United States with 18-year follow-up found that breakfast skipping in young adults (18–30 years old) was associated with reduced central obesity and obesity risk at middle-aged adulthood (36–48 years old) [46].

Earlier studies have proposed several mechanisms to explain why skipping breakfast can lead to obesity. Breakfast skipping is linked to improved appetite and diminished satiety. This could lead to over intake of food and insulin resistance [47]. Contrarily, breakfast consumption can help regulate hunger while enhancing insulin sensitivity for the next meal [20,47]. Breakfast eating can instantly interrupt fasting from the previous night. A more extended fasting period is associated with a higher ghrelin level, which can imitate fasting to improve human hedonic, orbitofrontal cortex, and hippocampal reactions to eating [48]. Obese adults skipping breakfast have a relative dietary compensation and higher calories consumption in subsequent meals during the day [49,50]. Furthermore, skipping breakfast may contribute to obesity by influencing genes expressions and hormones secretion. A study reported that skipping breakfast resulted in hyperglycemia after lunch and dinner, decreased intact glucagon-like peptide-1 (iGLP-1) level, and increased insulin resistance [51]. Breakfast skipping also influenced the expression of genes related to the circadian clock and metabolism, affecting circadian hormone production and raising postprandial blood glucose [52]. Another study showed that skipping breakfast is linked to stress-independent overactivity in the hypothalamic-pituitary-adrenal (HPA) axis, disrupting cortisol rhythm [53].

The absence of knowledge about the beneficial health effects of regular breakfast eating and limited time in the morning to prepare and eat breakfast are the main factors that lead to skipping breakfast, especially among young adults. Raising awareness about the health benefits of breakfast eating can positively impact breakfast-consumption frequency [54]. As a result, encouraging everyone to have breakfast regularly is a good recommendation for reducing obesity risk in primary prevention. Furthermore, it is suggested that future weight-loss programs include a recommendation about breakfast consumption [41,55].

The participants were recruited from public places, such as public gardens and shopping malls. These places are targeted frequently by adults from different countries living in Saudi Arabia for various reasons, such as shopping, relaxation, performing exercise, and eating foods. Thus, we believe that our sample was representative and accurately reflects the characteristics of the study population. Furthermore, persons with disabilities were excluded from this study because the nature of the disability could affect their body weight and height on one hand and their dietary behavior (particularly breakfast consumption) on the other. This effect could yield a bias in our results. Some limitations of this study could be addressed. Because of the cross-sectional design for this study, causal inference cannot be determined. Second, because no data on daily energy intake were available for analysis, we did not account for the influence of total calories eaten on BMI. Third, there is no standard definition for breakfast skipping in the scientific literature, generating bias. Lastly, women were not included in this study. Despite these limitations, a large multi-ethnic sample was used. Thus, the findings contribute to the body of evidence supporting the relevance of breakfast consumption in obesity prevention.

## 5. Conclusions

This study found that breakfast skipping was relatively high among a multi-ethnic population of young men living in Saudi Arabia. The results revealed significant variation in breakfast skipping rates among young men with various nationalities residing in Saudi Arabia. Moreover, the findings confirm a relationship between breakfast skipping and sociodemographic determinants and weight status. Further studies with robust designs and multi-ethnic samples and targeting different age and gender groups to prove the relationship between breakfast skipping and sociodemographic determinants and weight status are recommended.

## Figures and Tables

**Table 1:** Sociodemographic determinants and body weight status of study participants stratified according to breakfast-consumption patterns.

Variables	Breakfast Consumption (Eating Breakfast 7 Days/Week)	Breakfast Skipping (Eating Breakfast 0–6 Days/Week)	*p*-Value *
All Participants	1700 (47.2%)	1900 (52.8%)	
Nationality			˂0.001
Saudi	39 (13.5%)	250 (86.5%)	
Egyptian	107 (37.0%)	182 (63.0%)	
Yemeni	127 (37.9%)	208 (62.1%)	
Syrian	105 (35.8%)	188 (64.2%)	
Jordanian	46 (16.4%)	234 (83.6%)	
Sudanese	200 (72.5%)	76 (27.5%)	
Turkish	124 (61.1%)	79 (38.9%)	
Pakistani	153 (50.0%)	153 (50.0%)	
Afghan	190 (62.7%)	113 (37.3%)	
Indian	174 (58.6%)	123 (41.4%)	
Bangladeshi	301 (86.0%)	49 (14.0%)	
Filipino	134 (35.4%)	245 (64.6%)	
Age (years)	29.9 (3.2)	29.3 (3.2)	˂0.001
Residency Period in Saudi Arabia			0.064
1–5 years	1065 (48.5%)	1133 (51.5%)	
6 years or more	635 (45.3%)	767 (54.7%)	
Household Type			˂0.001
Non-family household	1490 (51.0%)	1430 (49.0%)	
Family household	210 (30.9%)	470 (69.1%)	
Marital Status			˂0.001
Single	789 (41.1%)	1130 (58.9%)	
Married	911 (54.2%)	770 (45.8%)	
Education Level			˂0.001
High school or less	1298 (56.8%)	986 (43.2%)	
College degree or more	402 (30.5%)	914 (69.5%)	
Monthly Income			˂0.001
Low (˂USD 1000)	1408 (53.5%)	1222 (46.5%)	
High (≥USD 1000)	292 (30.1%)	678 (69.9%)	
Body Mass Index (kg/m^2^)	24.8 (3.0)	25.4 (3.4)	˂0.001
Body Weight Status			˂0.001
Underweight/Normal weight	950 (51.1%)	910 (48.9%)	
Overweight/Obesity	750 (43.1%)	990 (56.9%)	

* Categorical variables were analyzed using chi-square test and expressed as numbers and percentages. Continuous variables were analyzed using independent samples *t*-test and expressed as means and standard deviations.

**Table 2:** Odds ratios of breakfast skipping among study participants for sociodemographic determinants and body mass index.

Variables	Unadjusted Odds Ratio *	95% CI	*p*-Value	Adjusted Odds Ratio **	95% CI	*p*-Value
Nationality						
Bangladeshi	1.00			1.00		
Saudi	39.38	25.04–61.93	**<0.001**	27.19	15.95–46.35	**<0.001**
Egyptian	10.45	7.11–15.35	**<0.001**	8.80	5.76–13.44	**<0.001**
Yemeni	10.06	6.92–14.62	**<0.001**	7.97	5.39–11.78	**<0.001**
Syrian	11.00	7.48–16.16	**<0.001**	9.22	5.97–14.25	**<0.001**
Jordanian	31.25	20.18–48.38	**<0.001**	29.10	17.97–47.11	**<0.001**
Sudanese	2.33	1.56–3.49	**<0.001**	2.23	1.48–3.37	**<0.001**
Turkish	3.91	2.59–5.92	**<0.001**	3.58	2.33–5.50	**<0.001**
Pakistani	6.14	4.22–8.95	**<0.001**	4.92	3.34–7.24	**<0.001**
Afghan	3.65	2.50–5.35	**<0.001**	3.02	2.04–4.47	**<0.001**
Indian	4.34	2.97–6.35	**<0.001**	4.10	2.79–6.03	**<0.001**
Filipino	11.23	7.77–16.23	**<0.001**	10.12	6.72–15.26	**<0.001**
Age (years)	0.95	0.93–0.97	**<0.001**	0.97	0.95–1.00	**0.044**
Residency Period in Saudi Arabia						
1–5 years	1.00			1.00		
6 years or more	1.14	0.99–1.30	0.064	1.10	0.92–1.31	0.292
Household Type						
Non-family household	1.00			1.00		
Family household	2.33	1.95–2.79	**<0.001**	0.89	0.69–1.16	0.400
Marital Status						
Single	1.00			1.00		
Married	0.59	0.52–0.67	**<0.001**	0.63	0.53–0.76	**<0.001**
Education Level						
High school or less	1.00			1.00		
College degree or more	2.99	2.59–3.46	**<0.001**	0.98	0.78–1.23	0.874
Monthly Income						
Low (˂USD 1000)	1.00			1.00		
High (≥USD 1000)	2.68	2.29–3.13	**<0.001**	1.37	1.10–1.71	**0.004**
Body Mass Index (kg/m^2^)	1.06	1.03–1.08	**<0.001**	1.03	1.01–1.06	**0.011**

* Univariate logistic regression analysis was used to test differences between breakfast skippers versus breakfast consumers (reference group). Differences were considered statistically significant at *p* value < 0.05, and significant values are presented in bold type. ** Multivariate logistic regression analysis was used to test differences between breakfast skippers versus breakfast consumers (reference group) after adjusting for subjects’ sociodemographic determinants and body mass index. Differences were considered statistically significant at *p*-value < 0.05, and significant values are presented in bold type.
